# *N*-Benzyl-2-(3-chloro-4-hy­droxy­phen­yl)acetamide

**DOI:** 10.1107/S1600536810035397

**Published:** 2010-09-08

**Authors:** Rohan A. Davis, Peter C. Healy

**Affiliations:** a Eskitis Institute for Cell and Molecular Therapies, Griffith University, Nathan, Brisbane 4111, Australia; b School of Biomolecular and Physical Sciences, Griffith University, Nathan, Brisbane 4111, Australia

## Abstract

The title compound, C_15_H_14_ClNO_2_, was synthesized as part of a project to generate a combinatorial library based on the fungal natural product 2-(3-chloro-4-hy­droxy­phen­yl)acetamide. It crystallizes as non-planar discrete mol­ecules [the peripheral 3-chloro-4-hy­droxy­phenyl and benzyl groups are twisted out of the plane of the central acetamide group, with N—C—C—C and C—C—C—C torsion angles of −58.8 (3) and 65.0 (2)°, respectively] linked by inter­molecular N—H⋯O and O—H⋯O hydrogen bonds.

## Related literature

For the preparation and characterization of the title compound, see: Poulsen *et al.* (2006 ▶); Davis *et al.* (2007 ▶). For the biological activity of the title compound, see: Davis *et al.* (2005 ▶, 2007 ▶). For background to organohalogen natural products, see: Gribble (1996 ▶). For related structures having the 3-chloro-4-hy­droxy­phenyl­acetamide moiety, see: Krohn *et al.* (1992 ▶); Davis *et al.* (2005 ▶); Davis & Healy (2008 ▶).
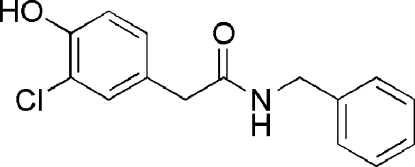


## Experimental

### 

#### Crystal data

C_15_H_14_ClNO_2_
*M* *_r_* = 275.72
Monoclinic, 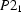

*a* = 4.8255 (2) Å
*b* = 10.8520 (5) Å
*c* = 12.7701 (6) Å
β = 96.055 (4)°
*V* = 664.99 (5) Å^3^
*Z* = 2
Mo *K*α radiation
μ = 0.28 mm^−1^
*T* = 296 K
0.55 × 0.40 × 0.04 mm

#### Data collection

Oxford-Diffraction Gemini S Ultra diffractometer
Absorption correction: multi-scan (*CrysAlis PRO*; Oxford Diffraction, 2010 ▶) *T*_min_ = 0.859, *T*_max_ = 0.989
4800 measured reflections
2334 independent reflections
1979 reflections with *I* > 2σ(*I*)
*R*_int_ = 0.020

#### Refinement

*R*[*F* ^2^ > 2σ(*F* ^2^)] = 0.030
*wR*(*F* ^2^) = 0.070
*S* = 0.96
2334 reflections
172 parameters
1 restraint
H-atom parameters constrained
Δρ_max_ = 0.13 e Å^−3^
Δρ_min_ = −0.15 e Å^−3^
Absolute structure: Flack (1983 ▶), 1098 Friedel pairs
Flack parameter: −0.11 (6)

### 

Data collection: *CrysAlis CCD* (Oxford Diffraction, 2009 ▶); cell refinement: *CrysAlis RED* (Oxford Diffraction, 2009 ▶); data reduction: *CrysAlis RED*; program(s) used to solve structure: *SIR97* (Altomare *et al.*, 1999 ▶); program(s) used to refine structure: *SHELXL97* (Sheldrick, 2008 ▶); molecular graphics: *ORTEP-3 for Windows* (Farrugia, 1997 ▶); software used to prepare material for publication: *PLATON* (Spek, 2009 ▶).

## Supplementary Material

Crystal structure: contains datablocks global, I. DOI: 10.1107/S1600536810035397/tk2709sup1.cif

Structure factors: contains datablocks I. DOI: 10.1107/S1600536810035397/tk2709Isup2.hkl

Additional supplementary materials:  crystallographic information; 3D view; checkCIF report

## Figures and Tables

**Table 1:** Hydrogen-bond geometry (Å, °)

*D*—H⋯*A*	*D*—H	H⋯*A*	*D*⋯*A*	*D*—H⋯*A*
N1—H1⋯O8^i^	0.86	2.15	2.9262 (18)	150
O4—H4⋯O8^ii^	0.92	1.85	2.767 (2)	180

Symmetry codes: (i) 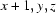; (ii) 
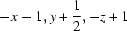
.

**Fig. 1.**
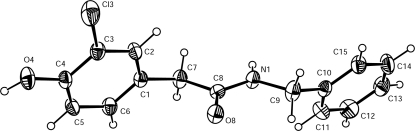
View of (I) with the atom numbering scheme. Displacement ellipsoids for non-H atoms are drawn at the 30% probability level.

**Fig. 2.**
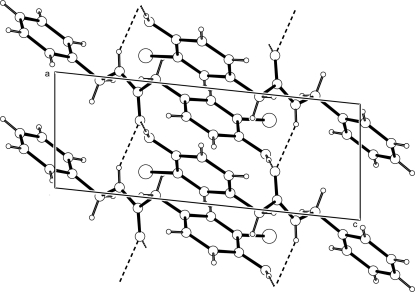
View down the crystallographic b axis of the hydrogen bonding arrangement (dashed lines) and crystal packing for (I).

## supplementary crystallographic information

### Comment

The title compound (I) (Fig. 1) was synthesized during the generation of a combinatorial library based on the fungal natural product 3-chloro-4-hydroxyphenylacetamide and was shown to display moderate cytotoxicity towards the human melanoma cell line MM96L and the human prostate cell line DU145 with IC_50_ values of 72 and 51 µ*M* respectively (Davis *et al.*, 2007). Although many organohalogen natural products have been identified (Gribble, 1996), only three crystal structures on compounds incorporating the 3-chloro-4-hydroxyphenylacetamide moiety have been reported to date (Krohn *et al.*, 1992; Davis *et al.*, 2005; Davis & Healy, 2008).

The present compound crystallizes in the chiral space group *P*2_1_ as discrete molecules with the central C1—C7—C8(-08)-N1—C9—C10 fragment approximately planar. The peripheral 3-chloro-4-hydroxyphenyl (C1—C7, O4, Cl3) and benzyl (C9—C15) groups are twisted out of the plane of the central acetamide group with N1—C9—C10—C11 and C2—C1—C7—C8 torsion angles of -58.8 (3) and 65.0 (2) °, respectively (Fig. 1). In the crystal lattice the amide (N1) and hydroxy (O4) groups form inter-molecular N—H···O and O—H···O hydrogen bonds with the carbonyl O atoms (O8) at (1 + *x*, *y*, *z*) and (-*x* - 1, *y* + 1/2, 1 - *z*), respectively (Table 1 & Fig. 2).

### Experimental

Compound (I) was prepared and analytically and spectroscopically characterized as previously reported (Davis *et al.*, 2007; Poulsen *et al.*, 2006). Crystals suitable for X-ray diffraction studies were obtained by recrystallization from a solution of the compound in a solvent mix of 90% methanol, 10% water, and 0.1% trifluoroacetic acid.

### Refinement

The carbon-bound H atoms were constrained as riding atoms with C—H = 0.93–0.96 Å. The amide and hydroxyl protons were located in difference Fourier maps and constrained with N—H 0.86 Å and O—H = 0.90 Å in the final refinement. *U*_iso_(H) values were set at 1.2*U*_eq_ of the parent atom.

### Crystal data

**Table:**

C_15_H_14_ClNO_2_	*F*(000) = 288
*M**_r_* = 275.72	*D*_x_ = 1.377 Mg m^−^^3^
Monoclinic, *P*2_1_	Mo *K*α radiation, λ = 0.71070 Å
Hall symbol: P 2yb	Cell parameters from 2771 reflections
*a* = 4.8255 (2) Å	θ = 3.2–32.2°
*b* = 10.8520 (5) Å	µ = 0.28 mm^−^^1^
*c* = 12.7701 (6) Å	*T* = 296 K
β = 96.055 (4)°	Plate, colourless
*V* = 664.99 (5) Å^3^	0.55 × 0.40 × 0.04 mm
*Z* = 2	

### Data collection

**Table:**

Oxford-Diffraction Gemini S Ultra diffractometer	2334 independent reflections
Radiation source: Enhance (Mo) X-ray Source	1979 reflections with *I* > 2σ(*I*)
graphite	*R*_int_ = 0.020
Detector resolution: 16.0774 pixels mm^-1^	θ_max_ = 25.0°, θ_min_ = 3.2°
ω and φ scans	*h* = −5→5
Absorption correction: multi-scan (*CrysAlis PRO*; Oxford Diffraction, 2010)	*k* = −12→12
*T*_min_ = 0.859, *T*_max_ = 0.989	*l* = −15→12
4800 measured reflections	

### Refinement

**Table:**

Refinement on *F*^2^	Secondary atom site location: difference Fourier map
Least-squares matrix: full	Hydrogen site location: inferred from neighbouring sites
*R*[*F*^2^ > 2σ(*F*^2^)] = 0.030	H-atom parameters constrained
*wR*(*F*^2^) = 0.070	*w* = 1/[σ^2^(*F*_o_^2^) + (0.0444*P*)^2^] where *P* = (*F*_o_^2^ + 2*F*_c_^2^)/3
*S* = 0.96	(Δ/σ)_max_ = 0.002
2334 reflections	Δρ_max_ = 0.13 e Å^−^^3^
172 parameters	Δρ_min_ = −0.15 e Å^−^^3^
1 restraint	Absolute structure: Flack (1983), 1098 Friedel pairs
Primary atom site location: structure-invariant direct methods	Flack parameter: −0.11 (6)

### Special details

**Table:**

Experimental. CrysAlisPro (Oxford Diffraction, 2010). Empirical absorption correction using spherical harmonics, implemented in SCALE3 ABSPACK scaling algorithm.
Geometry. Bond distances, angles *etc*. have been calculated using the rounded fractional coordinates. All su's are estimated from the variances of the (full) variance-covariance matrix. The cell e.s.d.'s are taken into account in the estimation of distances, angles and torsion angles
Refinement. Refinement on *F*^2^ for ALL reflections except those flagged by the user for potential systematic errors. Weighted *R*-factors *wR* and all goodnesses of fit *S* are based on *F*^2^, conventional *R*-factors *R* are based on *F*, with *F* set to zero for negative *F*^2^. The observed criterion of *F*^2^ > σ(*F*^2^) is used only for calculating -*R*-factor-obs *etc*. and is not relevant to the choice of reflections for refinement. *R*-factors based on *F*^2^ are statistically about twice as large as those based on *F*, and *R*-factors based on ALL data will be even larger.

### Fractional atomic coordinates and isotropic or equivalent isotropic displacement parameters (Å^2^)

**Table:**

	*x*	*y*	*z*	*U*_iso_*/*U*_eq_	
Cl3	−0.22153 (16)	0.10603 (6)	0.70233 (4)	0.0799 (3)	
O4	−0.5174 (3)	0.33833 (14)	0.69050 (12)	0.0612 (6)	
O8	−0.3412 (2)	0.08182 (14)	0.27899 (10)	0.0506 (5)	
N1	0.0633 (3)	0.05425 (16)	0.21183 (12)	0.0452 (5)	
C1	−0.1150 (4)	0.25973 (19)	0.42574 (15)	0.0386 (6)	
C2	−0.1047 (4)	0.1808 (2)	0.51082 (16)	0.0449 (7)	
C3	−0.2387 (4)	0.20842 (19)	0.59745 (15)	0.0452 (7)	
C4	−0.3886 (4)	0.31713 (18)	0.60254 (15)	0.0431 (7)	
C5	−0.3982 (4)	0.39584 (19)	0.51848 (17)	0.0480 (7)	
C6	−0.2645 (4)	0.36758 (19)	0.43020 (16)	0.0469 (7)	
C7	0.0273 (4)	0.2253 (2)	0.33001 (16)	0.0462 (7)	
C8	−0.0977 (3)	0.1137 (2)	0.27214 (13)	0.0366 (6)	
C9	−0.0319 (5)	−0.0518 (2)	0.14734 (18)	0.0568 (8)	
C10	0.1377 (4)	−0.06951 (19)	0.05671 (16)	0.0412 (7)	
C11	0.1558 (4)	0.0217 (2)	−0.01686 (18)	0.0577 (8)	
C12	0.3073 (5)	0.0053 (2)	−0.10153 (19)	0.0663 (9)	
C13	0.4390 (5)	−0.1045 (3)	−0.11432 (19)	0.0643 (9)	
C14	0.4229 (5)	−0.1963 (2)	−0.04274 (19)	0.0660 (9)	
C15	0.2735 (5)	−0.1792 (2)	0.04367 (18)	0.0531 (8)	
H1	0.23170	0.07840	0.20990	0.0540*	
H2	−0.00440	0.10590	0.50930	0.0540*	
H4	−0.56440	0.41910	0.70050	0.0730*	
H5	−0.49940	0.47080	0.52080	0.0570*	
H6	−0.27380	0.42290	0.37290	0.0580*	
H11	0.06190	0.09810	−0.00830	0.0710*	
H12	0.31640	0.06970	−0.15180	0.0840*	
H13	0.54640	−0.11580	−0.17020	0.0790*	
H14	0.50880	−0.27320	−0.05200	0.0800*	
H15	0.26570	−0.24260	0.09380	0.0660*	
H71	0.01580	0.29320	0.28310	0.0550*	
H72	0.21760	0.20810	0.35260	0.0550*	
H91	−0.22280	−0.03950	0.12110	0.0690*	
H92	−0.01890	−0.12390	0.19040	0.0690*	

### Atomic displacement parameters (Å^2^)

**Table:**

	*U*^11^	*U*^22^	*U*^33^	*U*^12^	*U*^13^	*U*^23^
Cl3	0.1357 (6)	0.0590 (4)	0.0499 (3)	0.0134 (4)	0.0333 (3)	0.0106 (3)
O4	0.0733 (10)	0.0631 (10)	0.0523 (9)	0.0062 (8)	0.0312 (8)	−0.0102 (8)
O8	0.0325 (6)	0.0649 (11)	0.0572 (8)	−0.0121 (6)	0.0183 (6)	−0.0131 (7)
N1	0.0279 (7)	0.0588 (11)	0.0510 (10)	−0.0130 (7)	0.0142 (7)	−0.0190 (9)
C1	0.0343 (9)	0.0426 (11)	0.0406 (11)	−0.0076 (8)	0.0115 (8)	−0.0073 (9)
C2	0.0506 (11)	0.0393 (12)	0.0464 (12)	0.0077 (9)	0.0128 (9)	−0.0042 (10)
C3	0.0564 (12)	0.0425 (11)	0.0382 (11)	−0.0023 (9)	0.0124 (10)	−0.0009 (9)
C4	0.0439 (11)	0.0464 (12)	0.0409 (11)	0.0001 (9)	0.0140 (9)	−0.0086 (10)
C5	0.0495 (12)	0.0446 (12)	0.0515 (13)	0.0108 (9)	0.0133 (10)	−0.0059 (11)
C6	0.0550 (12)	0.0431 (12)	0.0440 (12)	−0.0021 (10)	0.0122 (10)	−0.0004 (10)
C7	0.0428 (10)	0.0524 (13)	0.0468 (11)	−0.0101 (10)	0.0201 (9)	−0.0079 (10)
C8	0.0304 (9)	0.0481 (11)	0.0325 (9)	−0.0038 (9)	0.0095 (7)	0.0021 (9)
C9	0.0489 (12)	0.0657 (15)	0.0591 (14)	−0.0192 (10)	0.0209 (11)	−0.0221 (12)
C10	0.0353 (10)	0.0465 (13)	0.0426 (11)	−0.0105 (9)	0.0075 (8)	−0.0099 (10)
C11	0.0580 (13)	0.0485 (14)	0.0683 (16)	0.0089 (11)	0.0151 (12)	0.0015 (12)
C12	0.0759 (16)	0.0680 (17)	0.0571 (15)	−0.0015 (13)	0.0172 (13)	0.0140 (13)
C13	0.0702 (15)	0.0805 (19)	0.0456 (13)	−0.0025 (13)	0.0221 (11)	−0.0122 (13)
C14	0.0833 (18)	0.0581 (16)	0.0593 (15)	0.0144 (12)	0.0209 (13)	−0.0156 (13)
C15	0.0693 (14)	0.0426 (13)	0.0482 (12)	−0.0012 (10)	0.0099 (10)	−0.0025 (10)

### Geometric parameters (Å, °)

**Table:**

Cl3—C3	1.736 (2)	C10—C11	1.374 (3)
O4—C4	1.360 (2)	C11—C12	1.379 (3)
O8—C8	1.2368 (18)	C12—C13	1.368 (4)
O4—H4	0.9200	C13—C14	1.360 (4)
N1—C8	1.319 (2)	C14—C15	1.393 (3)
N1—C9	1.461 (3)	C2—H2	0.9500
N1—H1	0.8600	C5—H5	0.9500
C1—C7	1.511 (3)	C6—H6	0.9400
C1—C6	1.379 (3)	C7—H71	0.9500
C1—C2	1.380 (3)	C7—H72	0.9500
C2—C3	1.372 (3)	C9—H91	0.9600
C3—C4	1.389 (3)	C9—H92	0.9500
C4—C5	1.369 (3)	C11—H11	0.9600
C5—C6	1.391 (3)	C12—H12	0.9500
C7—C8	1.511 (3)	C13—H13	0.9300
C9—C10	1.499 (3)	C14—H14	0.9400
C10—C15	1.377 (3)	C15—H15	0.9400
			
Cl3···O4	2.8932 (17)	H1···O8^vii^	2.1500
Cl3···C12^i^	3.554 (2)	H1···C11	2.9500
Cl3···H12^ii^	3.0800	H1···H72	2.3100
Cl3···H12^i^	3.0600	H1···H4^viii^	2.5500
Cl3···H15^iii^	3.1000	H2···C8	3.0200
O4···Cl3	2.8932 (17)	H2···C5^viii^	3.0400
O4···O8^iv^	2.767 (2)	H2···C6^viii^	2.9600
O4···C9^iv^	3.375 (3)	H4···H5	2.4100
O4···C9^iii^	3.403 (3)	H4···O8^iv^	1.8500
O8···O4^v^	2.767 (2)	H4···N1^iii^	2.9500
O8···N1^vi^	2.9262 (18)	H4···C8^iv^	2.7100
O8···C2	3.242 (2)	H4···C9^iv^	2.9100
O4···H92^iii^	2.8900	H4···H1^iii^	2.5500
O4···H92^iv^	2.8600	H5···H4	2.4100
O8···H72^vi^	2.7800	H5···C2^iv^	2.9700
O8···H91	2.5200	H6···H71	2.3600
O8···H1^vi^	2.1500	H11···N1	2.8500
O8···H4^v^	1.8500	H11···C15^xii^	2.9200
N1···O8^vii^	2.9262 (18)	H11···H14^x^	2.5500
N1···H11	2.8500	H11···H15^xii^	2.5100
N1···H4^viii^	2.9500	H12···Cl3^ix^	3.0600
C2···O8	3.242 (2)	H12···Cl3^xiii^	3.0800
C9···O4^viii^	3.403 (3)	H14···C11^xiv^	2.8400
C9···O4^v^	3.375 (3)	H14···H11^xiv^	2.5500
C12···Cl3^ix^	3.554 (2)	H15···H92	2.3300
C2···H5^v^	2.9700	H15···Cl3^viii^	3.1000
C5···H2^iii^	3.0400	H15···H11^xi^	2.5100
C6···H2^iii^	2.9600	H71···H6	2.3600
C8···H4^v^	2.7100	H72···O8^vii^	2.7800
C8···H2	3.0200	H72···H1	2.3100
C9···H4^v^	2.9100	H91···O8	2.5200
C11···H1	2.9500	H91···C14^vi^	3.0700
C11···H14^x^	2.8400	H91···C15^vi^	2.9500
C14···H91^vii^	3.0700	H92···H15	2.3300
C15···H91^vii^	2.9500	H92···O4^v^	2.8600
C15···H11^xi^	2.9200	H92···O4^viii^	2.8900
			
C4—O4—H4	115.00	C10—C15—C14	120.2 (2)
C8—N1—C9	123.03 (16)	C1—C2—H2	119.00
C8—N1—H1	119.00	C3—C2—H2	119.00
C9—N1—H1	118.00	C4—C5—H5	119.00
C2—C1—C6	118.01 (18)	C6—C5—H5	120.00
C2—C1—C7	120.12 (18)	C1—C6—H6	119.00
C6—C1—C7	121.85 (18)	C5—C6—H6	120.00
C1—C2—C3	121.21 (19)	C1—C7—H71	109.00
C2—C3—C4	121.02 (19)	C1—C7—H72	108.00
Cl3—C3—C2	119.61 (16)	C8—C7—H71	109.00
Cl3—C3—C4	119.37 (15)	C8—C7—H72	108.00
O4—C4—C3	117.91 (17)	H71—C7—H72	110.00
O4—C4—C5	124.18 (18)	N1—C9—H91	109.00
C3—C4—C5	117.91 (18)	N1—C9—H92	109.00
C4—C5—C6	121.19 (19)	C10—C9—H91	109.00
C1—C6—C5	120.66 (19)	C10—C9—H92	109.00
C1—C7—C8	113.70 (16)	H91—C9—H92	109.00
O8—C8—C7	121.53 (16)	C10—C11—H11	119.00
O8—C8—N1	121.81 (18)	C12—C11—H11	120.00
N1—C8—C7	116.64 (15)	C11—C12—H12	120.00
N1—C9—C10	111.81 (18)	C13—C12—H12	120.00
C9—C10—C11	121.00 (19)	C12—C13—H13	121.00
C9—C10—C15	120.65 (19)	C14—C13—H13	120.00
C11—C10—C15	118.33 (19)	C13—C14—H14	120.00
C10—C11—C12	121.4 (2)	C15—C14—H14	119.00
C11—C12—C13	119.8 (2)	C10—C15—H15	120.00
C12—C13—C14	119.8 (2)	C14—C15—H15	120.00
C13—C14—C15	120.4 (2)		
			
C8—N1—C9—C10	156.64 (18)	C3—C4—C5—C6	0.6 (3)
C9—N1—C8—O8	1.0 (3)	O4—C4—C5—C6	−179.11 (18)
C9—N1—C8—C7	−177.14 (17)	C4—C5—C6—C1	−0.9 (3)
C6—C1—C2—C3	0.0 (3)	C1—C7—C8—O8	23.7 (3)
C7—C1—C2—C3	−178.18 (18)	C1—C7—C8—N1	−158.08 (17)
C2—C1—C6—C5	0.6 (3)	N1—C9—C10—C11	−58.8 (3)
C6—C1—C7—C8	−113.1 (2)	N1—C9—C10—C15	122.9 (2)
C2—C1—C7—C8	65.0 (2)	C9—C10—C15—C14	177.8 (2)
C7—C1—C6—C5	178.69 (18)	C11—C10—C15—C14	−0.5 (3)
C1—C2—C3—C4	−0.2 (3)	C9—C10—C11—C12	−178.8 (2)
C1—C2—C3—Cl3	179.67 (16)	C15—C10—C11—C12	−0.5 (3)
Cl3—C3—C4—O4	−0.2 (3)	C10—C11—C12—C13	1.2 (3)
C2—C3—C4—C5	−0.1 (3)	C11—C12—C13—C14	−0.9 (4)
Cl3—C3—C4—C5	−179.95 (15)	C12—C13—C14—C15	−0.1 (4)
C2—C3—C4—O4	179.67 (18)	C13—C14—C15—C10	0.8 (4)

Symmetry codes: (i) *x*, *y*, *z*+1; (ii) *x*−1, *y*, *z*+1; (iii) −*x*, *y*+1/2, −*z*+1; (iv) −*x*−1, *y*+1/2, −*z*+1; (v) −*x*−1, *y*−1/2, −*z*+1; (vi) *x*−1, *y*, *z*; (vii) *x*+1, *y*, *z*; (viii) −*x*, *y*−1/2, −*z*+1; (ix) *x*, *y*, *z*−1; (x) −*x*+1, *y*+1/2, −*z*; (xi) −*x*, *y*−1/2, −*z*; (xii) −*x*, *y*+1/2, −*z*; (xiii) *x*+1, *y*, *z*−1; (xiv) −*x*+1, *y*−1/2, −*z*.

### Hydrogen-bond geometry (Å, °)

**Table:**

*D*—H···*A*	*D*—H	H···*A*	*D*···*A*	*D*—H···*A*
N1—H1···O8^vii^	0.86	2.15	2.9262 (18)	150
O4—H4···O8^iv^	0.92	1.85	2.767 (2)	180

Symmetry codes: (vii) *x*+1, *y*, *z*; (iv) −*x*−1, *y*+1/2, −*z*+1.
